# Fatty Acid Content of Four Salmonid Fish Consumed by Indigenous Peoples from the Yamal-Nenets Autonomous Okrug (Northwestern Siberia, Russia)

**DOI:** 10.3390/ani12131643

**Published:** 2022-06-27

**Authors:** Alexander G. Dvoretsky, Fatima A. Bichkaeva, Olga S. Vlasova, Sergei V. Andronov, Vladimir G. Dvoretsky

**Affiliations:** 1 Murmansk Marine Biological Institute of the Russian Academy of Sciences (MMBI RAS), 183010 Murmansk, Russia; ag-dvoretsky@yandex.ru; 2 N. Laverov Federal Center for Integrated Arctic Research of the Ural Branch of the Russian Academy of Sciences (FECIAR UrB RAS), 163000 Arkhangelsk, Russia; fatima@fciarctic.ru (F.A.B.); olgawlassova@mail.ru (O.S.V.); 3 National Medical Research Center for Rehabilitation of Balneology, 121099 Moscow, Russia; sergius198010@mail.ru

**Keywords:** fatty acids, Arctic cisco, least cisco, muksun, Arctic charr, northwestern Siberia, traditional diets

## Abstract

**Simple Summary:**

Fish is an important source of nutritious fatty acids, which are necessary for the prevention of cardiovascular and neurological diseases. Many fish species are a major component of the human diet, especially for indigenous peoples from Siberia, Russia, but information about their fatty acid profiles is scarce. We studied the fatty acid content of four salmonid fish (Arctic cisco, least cisco, muksun, and Arctic charr) inhabiting water bodies of the Gydan Peninsula, Kara Sea basin. We found a high content of essential polyunsaturated fatty acids in local salmonid fishes. Arctic charr was the fish species with the highest concentration of fatty acids in the flesh. The results of our multivariate analysis may be useful for anglers, stakeholders, and local consumers.

**Abstract:**

We assayed fatty acids in the flesh of Arctic cisco *Coregonus autumnalis* (adult and juvenile), least cisco *Coregonus sardinella*, muksun *Coregonus muksun*, and Arctic charr *Salvelinus alpinus* inhabiting water bodies of the Gydan Peninsula, Siberia, Russia. The highest concentrations of total and polyunsaturated fatty acids (PUFAs) were found in Arctic charr (27.8 and 9.5 mg g^−1^) and adult Arctic cisco (20.2 and 7.6 mg g^−1^), while the lowest concentrations occurred in juvenile Arctic cisco (7.5 and 3.6 mg g^−1^). Multivariate analyses divided all studied fish into five distinct groups with the highest similarity between least cisco and muksun and the highest dissimilarity between juvenile Arctic cisco and Arctic charr. Coregonid fish from the study area had a higher content of docosahexaenoic and eicosapentaenoic acids than their conspecifics from subarctic and temperate habitats. The flesh of the studied fish is a source of a healthy diet for humans. Taking into account that all the studied fish are components of the traditional diet of indigenous peoples in northwestern Siberia, our data may be useful not only for local consumers and anglers but also for stakeholders focused on food policy and food security in the area.

## 1. Introduction

The Yamal-Nenets Autonomous Okrug (district) (YNAO) is located in northwest Siberia, stretching from the Ural Mountains northwards into the Kara Sea. This region covers a large territory of 769,250 km^2^, more than half of which is situated above the Arctic Circle [1]. YNAO encompasses the largest wetland in the world with around 48 thousand rivers and 3–5 thousand lakes. The region is very scarcely populated (a total population of 553,000), with an average of 0.7 person km^−2^.

The ethnic groups who maintain their traditional lifestyles and have to adapt to the harsh conditions of the Arctic (10% of the total population) strongly depend on the consumption of traditional products [2], among which local fish species play one of the most important roles, especially during the winter period. At the present time, high consumption of traditional foods is typical only for the season of fishing or reindeer slaughter. In contrast, out of season, the consumption of foods rich in easily digestible carbohydrates (sugar, white bread, gingerbread, and condensed milk) increases [3].

Salmonid fish are rich in fat and, therefore, are considered important food sources for indigenous inhabitants of Western Siberia’s Arctic zone. Among these fish, the most important contributors to the traditional fish diets are the Arctic cisco *Coregonus autumnalis* (Pallas, 1776), the least cisco *Coregonus sardinella* Valenciennes, 1848, and the muksun *Coregonus muksun* (Pallas, 1814). The two former species are semi-anadromous, i.e., they spend adult life in low to moderate salinity habitats with migrations into upstream freshwater rivers and floodplains for spawning, while the latter species is freshwater [4]. Another semi-anadromous species, the Arctic charr *Salvelinus alpinus* (Linnaeus, 1758), is a desirable target for traditional fishing, but it is less abundant than coregonids. 

In the YNAO, there have been negative trends in the catch dynamics of salmonid fish species in the past decades. For example, in 1999, the total catch of *Coregonus muksun* accounted for 1257 t; it declined to 310–590 t in the 2000s and 49 t in 2014. During the period from the mid-1990s to the early 2000s, the annual illegal catch amounted to 800–1500 t [5]. In 2014, a ban on commercial muksun fisheries was established to prevent further depletion of the stock. In 2020, the official landing was approximately 1 t for aquaculture and scientific purposes only. The rate of illegal fishery remained extremely high, averaging 116 t in 2020 [6]. In the YNAO, Arctic cisco has been captured since 1941. From the 1970s to the 1990s, the total catch rates were quite stable, accounting for 150 t per year. They dropped to 50 t per year in the 2000s and increased again to 100 t in the mid-2010s. In 2020, the total annual catch was 44 t [6], and the illegal catch rate was estimated to be 20 t. During the period from 1995 to 2007, the average annual catch of *Coregonus sardinella* decreased from 337 to 181 t, and currently, this species is not an object for commercial fishing [7], but it is fished by indigenous peoples and anglers.

Thus, the indigenous peoples living on the northern coast of the Gydan Peninsula and other locations of West Siberian Plain have to change their diets, reacting to the availability of the mentioned salmonid fishes. To formulate new health rations, information about fatty acid profiles of local fish species is required. In Siberia, previous studies have been focused on salmonid fish from some subarctic and arctic lakes and from the Yenisey River, i.e., continental water bodies [8,9], while information concerning fish from water bodies immediately adjacent to the Kara Sea is lacking.

For this reason, our paper was aimed to study fatty acid profiles of Arctic cisco, least cisco, muksun, and Arctic charr from the Gyda River and Gydan Bay of the Kara Sea basin.

## 2. Materials and Methods

### 2.1. Study Area and Sampling

**Figure 1:**
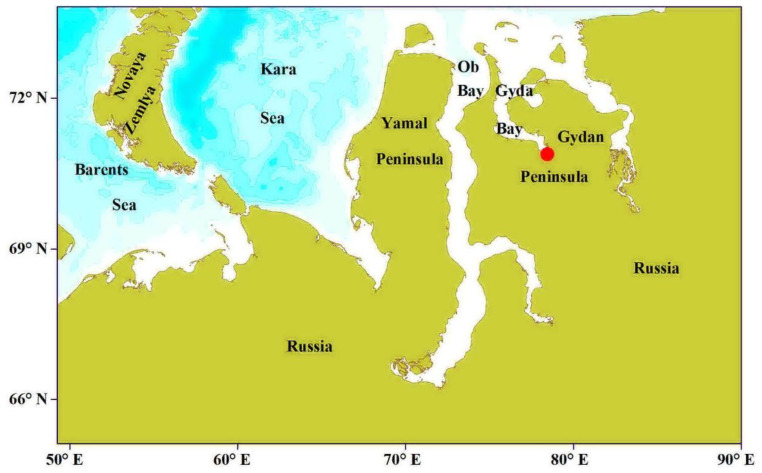
Location of the sampling area in the Gydan Peninsula, Russian Arctic.

Fish were obtained from local authorized fishers just after being caught in water bodies located near the Gyda settlement (70°53′41″ N, 78°30′14″ E, northern coast of the Gydan Peninsula, Figure 1). 

The study area is located in the northern part of the West Siberian plain, between the Gulf of Taz and Ob Bay in the west and the Yenisei Bay in the east. This zone can be characterized by having a harsh arctic climate with average January temperatures ranging from −24 to −28 °C and average July temperatures ranging from 5 to 10.2 °C. Snow coverage occurs from September until early June [10].

The samples were taken from four fish species: Arctic cisco, least cisco, muksun, and Arctic charr (Table 1). 

Adult and juvenile specimens of Arctic cisco were considered and analyzed separately. For biochemical analyses, samples of white muscle tissue of approximately 0.1–0.5 g were taken 1–2 cm below the dorsal fin. The samples were taken accurately to avoid skin, red muscle, and bones. These samples were frozen and then transferred to the laboratory of the Federal Center for Integrated Arctic Research (Arkhangelsk) for fatty acid analyses. Lipid analyses were performed within 2–3 months after sampling.

### 2.2. Biochemical Assay

The lipid fraction was first extracted from fish samples by the Folch method [11] with modifications [12]. A detailed description is provided in our previous papers [13,14]. Briefly, the homogenized sample was transferred into a 100-mL glass flask containing 10 mL of a chloroform-methanol mixture (2:1) and a solution of nonadecanoic acid (0.2 mg). The resulting solution was mixed for 30 min and transferred to a thermostat at 25 °C for 10–12 h. The solution was filtered into a tube, and the mixture of chloroform–methanol was added. Three ml of 0.74% of water solution of CaCl_2_ were added to the tube and placed in a refrigerator for 12 h. After stratification, the lower layer was used for further analysis. A total of 0.5–1.0 mL of methanol was added to the vial, and the contents were evaporated to dryness using a vacuum evaporator “Multivopar” (BÜCHI Labortechnik AG, Flawil, Switzerland). The evaporated extract was dissolved in 0.2 mL of the chloroform–methanol mixture and mixed for 5 min. Then 2 mL of a mixture containing 1.5% solution of H_2_SO_4_ in methanol was added to the tube, and the sample was incubated for 30 min at 90 °C in a water bath. At the end of incubation, the sample was transferred to a 10-mL tube containing 0.8 mL of distilled water and incubated at ambient temperature for 2–4 h. The upper fraction was transferred to a 2-mL vial and evaporated. Fatty acid methyl esters were analyzed using a 7890A gas chromatograph (Agilent Technologies Inc., Wilmington, DE, USA) with a flame ionization detector and a capillary column Agilent DB-23; 60 m × 0.25 mm × 0.15 μm (Agilent Technologies Inc., Santa Clara, CA, USA). Nitrogen was used as the carrier gas at a flow rate of 1 mL min^−1^. An aliquot of the sample (200 μL) was injected under the following conditions: the oven temperature started at 130 °C; it was then increased to 170 °C at a rate of 8.5 °C min^−1^, before being once again increased to 206 °C at 2 °C min^−1^ and then to 220 °C at a rate of 0.7 °C min^−1^; finally, the temperature was increased to 220 °C with a rate of 6 °C min^−1^. The injector temperature was set at 270 °C, and the detector temperature was set at 280 °C. Individual fatty acid methyl esters were identified by comparison with a standard mixture of methyl esters of fatty acids Nu Chek Prep Inc 569 B (Nu Chek Prep Inc., Elysian, MN, USA) using the Agilent Chem Station B.04.03 software (Agilent Technologies Inc., Waldbronn, Germany).

The fatty acid concentrations were used to calculate the atherogenic (*AI*), thrombogenic (*TI*), and *h*/*H* indices as follows [15]:

$$AI=\frac{C12:0+4\cdot C14:0+C16:0}{\sum MUFA+\sum n3+\sum n6}$$

$$TI=\frac{C14:0+C16:0+C18:0}{0.5\cdot \sum MUFA+0.5\cdot \sum n6+3\cdot \sum n3+\frac{\sum n3}{\sum n6}}$$

$$h/H=\frac{C18:1+C18:2+C18:3+C20:1+C22:1+C24:1}{C14:0+C16:0}$$

where ∑*MUFA* = total monounsaturated fatty acids, *n*3 = total omega-3 PUFA, *n*6 = total omega-6 PUFA.

### 2.3. Statistical Analysis

Fatty acid concentrations in relation to fish species were compared using a one-way analysis of variance (ANOVA) followed by Tukey’s HSD post hoc test. Before statistical analyses, fatty acid data were checked for normal distribution and variance homogeneity using the Shapiro–Wilk test and modified Levene’s test, and then the fatty acid data were square-root transformed to ensure the assumptions required for parametric analyses.

Multivariate analyses, including classification (cluster analysis) by hierarchical agglomerative clustering with group-average linkage and ordination by non-metric multidimensional scaling (NMDS) [16], were used to reveal the degree of similarity in fatty acid signatures among the fish species studied. The Bray–Curtis similarity index, a metric capable of handling joint absences robustly, and considers zero as a lack of data rather than a value [17,18], was used as the basis for cluster analysis and NMDS. The cluster analysis is a method that interconnects samples by their associations, creating a dendrogram in which similar samples to the chosen variables are grouped together. The ordination plot is another way to visualize these similarities. NMDS ordination represents samples as points in a 2D plane. 

The SIMPER routine was applied to summarize which fatty acids contribute to the dissimilarity between species groupings. Differences in the total fatty concentrations were tested with analysis of similarity (ANOSIM) permutation tests [19] to assess whether the fatty acid profiles varied between the groups delineated with cluster analysis. The two-way ANOSIM used a resemblance matrix based on the Bray–Curtis similarity of transformed fatty acid data in our study and tested the null hypothesis that there were no differences in similarity among fatty acids between the fish species. Statistical analyses were performed by using Primer Version 5.0 (Plymouth Routines in Multivariate Ecological Research, Plymouth, UK) for Windows and STATISTICA 10. The data are presented as means ± SE (standard errors).

## 3. Results

A total of 43 fatty acids were identified in the fish samples, among which 28 fatty acids each exceeded 0.1% of the total content (Table 2). 

The main saturated fatty acids (SFA) were palmitic (C16:0) and stearic (C18:0) acids. The concentration of C16:0 varied from 1720 μg g^−1^ in Arctic cisco juveniles to 5400 μg g^−1^ in Arctic charr. The minimum and maximum levels of C18:0 (391 and 1590 μg g^−1^) were registered in the same species. Oleic (C18:1n9c) and palmitoleic (C16:1n7c) acids were the dominant monounsaturated fatty acids (MUFA) ranging from 850 to 6340 μg g^−1^ and 350 to 2810 μg g^−1^, respectively. Juvenile Arctic cisco and Arctic charr demonstrated the lowest and the highest values, respectively. A total of 15 polyunsaturated fatty acids (PUFA) were found in salmonid fish. Arachidonic acid (C20:4n6) was the major n-6 PUFA accounting for 260–669 μg g^−1^. The most abundant n-3 PUFAs were docosahexaenoic acid (DHA, C22:6n3) and eicosapentaenoic acid (EPA, C20:5n3). DHA ranged from 1900 μg mg^−1^ in juvenile Arctic cisco to 4310 μg g^−1^ in Arctic charr, while EPA varied from 755 to 2110 μg g^−1^ in juvenile and adult Arctic cisco, respectively. Fatty acid signatures differed among the fish species (Table 2). Arctic cisco (both juvenile and adult) and least cisco contained a higher proportion of PUFAs than that of MUFAs and SFAs, while muksun and Arctic charr demonstrated approximately equal proportions of different fatty acid categories (Table 2).

All fish species were rich in essential n-3 PUFAs; the ∑n-3/∑n-6 ratio varied from 3.14 in Arctic charr to 4.87 in juvenile Arctic cisco and least cisco. As a result, the atherogenic (AI) and thrombogenic (TI) indices were low, with the minimum levels in Arctic charr and least cisco, respectively. The ratio between hypocholesterolemic and hypercholesterolemic fatty acids (h/H index) ranged from 1.89 in muksun to 2.38 in the least cisco (Table 2).

**Figure 2:**
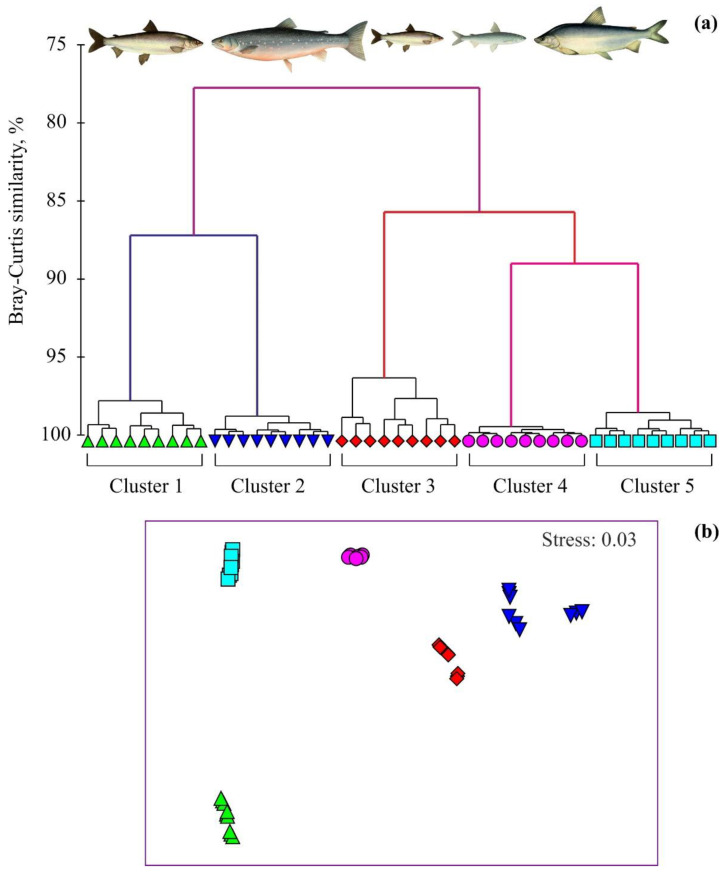
Dendrogram of hierarchical cluster analysis (group average method) (**a**) and nMDS plot (**b**) of fatty-acid profiles (square-root-transformed relative percentages; Bray–Curtis similarities) of fish species in the Gyda River, Russian Arctic. Cluster 1—Arctic cisco *Coregonus autumnalis* (adult specimens), Cluster 2—Arctic charr *Salvelinus alpinus*, Cluster 3—Arctic cisco *Coregonus autumnalis* (juvenile specimens), Cluster 4—least cisco *Coregonus sardinella*, Cluster 5—muksun *Coregonus muksun*.

Multivariate analyses (hierarchical cluster analysis and NMDS ordination) of the fatty acid composition separated the fish into five distinct groups (Figure 2a), which corresponded to adult Arctic cisco, Arctic charr, juvenile Arctic cisco, least cisco, and muksun, respectively. 

Fatty acid compositions of fish species were distinct from one to another according to the nMDS plot (*p* < 0.001; Figure 2b). The ANOSIM indicated that there were significant differences in fatty acids for the five groupings delineated with the cluster analysis and NMDS (global R between sample = 0.807, *p* < 0.001, 999 permutations). Pair-wise comparisons also demonstrated significant differences in fatty acid profiles among the fish species with the highest dissimilarity for the pair Arctic cisco (juvenile)—Arctic charr (Table S1). 

SIMPER revealed that most percentages of the fatty acid dissimilarity within fish species were mainly driven by variations in seven major fatty acids (C16:1n7, C18:1n9, C14:0, C16:0, C18:3n3, C22:5n3, and C22:6n3), but in some cases, C18:1n9, C18:0, C18:2n6, and C20:5n3 also contributed to dissimilarities (Table S2).

## 4. Discussion

Previous research on salmonid fish from water bodies located in the Arctic and subarctic zones of Siberia indicated significant variations in their fatty acid signatures [8,9]. Our results conducted in the northern part of the West Siberian plain confirmed this pattern: although the congeneric species are genetically close to each other, their fatty acid contents demonstrated substantial fluctuations. Moreover, within the same species (Arctic cisco), we found a high level of dissimilarity in different-sized fish, with the flesh of adult specimens containing two-to–eight-fold higher concentrations of major fatty acids than juveniles. Most likely, this result is associated with a change in feeding habits during growth. A similar result was reported by Yoshii et al. [20], who studied ontogenic diet changes in *Coregonus autumnalis migratorius* from Lake Baikal.

The fatty acid profiles in the studied adult salmonid fish displayed remarkable spatial variations among habitats situated in the Arctic, subarctic, and temperate zones [21,22,23] (Figure S1). For instance, Arctic cisco from the Gyda River had a higher MUFA but a lower PUFA level than in Lake Baikal (temperate zone, southern part of West Siberia). Furthermore, the total fatty acid concentration in this fish from the Gyda River was five times lower than in the Yenisei River situated in the subarctic zone (20.2 vs. 100.3 mg g^−1^) [8]. The least cisco had the same total levels of fatty acids (11.3 mg g^−1^) in Arctic habitats (the Gyda River and Lake Sobachye), but this concentration was five times lower than that reported for *C. sardinella* from the Yenisei River [8]. At the same time, the percentages of EPA and DHA were higher in the fish from the Gyda River in comparison to the Yenisei River. Arctic charr is known to have especially high morphological and ecological diversity [23] with normal, small, and dwarf forms. It is widely distributed at high latitudes. In our study area, Arctic charr had a higher percentage content of oleic acid but lower levels of EHA and DHA than reported for other locations [22]. The total absolute concentration of fatty acids in Arctic charr from the Gyda River (27.8 mg g^−1^) exceeded the levels found in an Arctic lake (Lake Sobachye, 3.4 mg g^−1^), in temperate Lakes Tokko and Leprindo (ca. 10 mg g^−1^), and in a subarctic Lake Ladoga (ca. 10 mg g^−1^) [22].

The spatial variations of fatty acid profiles may be explained by differences in environmental conditions in the mentioned water bodies. As fatty acids are involved in cell metabolism and structure, and a degree of saturation of phospholipid fatty acids correlates to cell membrane fluidity, fluctuations in water temperature may lead to changes in the content of fish fatty acids [24,25]. Furthermore, DHA and EPA have been shown to preferentially incorporate phospholipids at cold temperatures [26]. Experimental studies showed that decreasing water temperature leads to increased unsaturated fatty acid proportions with a corresponding decrease in SFA and an increase in PUFA as part of homeoviscous adaptation to maintain fluidity [27,28,29]. This pattern can explain the higher proportions of PUFAs in the flesh of our coregonid fish in comparison to individuals from subarctic and temperate habitats. In the case of Arctic charr, we registered an opposite pattern: the levels of DHA and EPA were lower in the Gyda River, indicating that other factors may contribute to this result. Indeed, nutrition, in some cases, plays a more important role in driving fatty acid signatures in fish [30,31]. However, in most cases, it is a combination of both nutrition and temperature that has the greatest influence on the fatty acid composition in fish [32,33]. 

In our study area, adult individuals of Arctic charr feed on other fish, including smaller conspecifics and least cisco [34]. A similar diet is described for this species in Lake Sobachye, where it consumes *Coregonus tugun* and *C. sardinella* [22]. In Lake Tokko and Lake Lepindo, Arctic charr is presented by small and dwarf forms ingesting the lake minnow *Phoxinus percnurus* (fish) and zooplankton + Chironomidae, respectively [22]. Interestingly, the amphipod *Pontoporeia affinis* is the main food item for muksun and least cisco in the Gyda River and in the Yenisei River [35,36,37]. This can explain the high similarity found between their fatty acid profiles in the study area (Figure 2).

In fish, many fatty acids cannot be biosynthesized de novo or can be synthesized from their precursors to a limited extent, with actual rates of fatty acid synthesis de novo being inversely related to the level of lipid in the diet [38]. Therefore, fish usually obtain fatty acids from dietary supplements [39]. Thus, different feeding habits may explain the spatial variations in the fatty acid signatures found in salmonid fish of the same species. Spatial variations in fatty acid profiles of aquatic organisms under different habitats and feeding conditions are well-known in freshwater and marine environments [40,41,42].

Fish products, especially flesh, have long been recognized as important sources of low-fat protein and essential fatty acids, particularly n-3 and n-6 PUFAs, among which EPA and DHA are the most important [9,31]. Many previous studies have demonstrated that PUFAs can act as a preventative measure against cardiovascular disorders and hypertension in adults and are vital in fetal brain development [43,44]. The consumption of n-3 PUFAs also has positive effects on ocular health [45] and contributes to preventing arthritis and other inflammatory disorders [46,47,48] as well as neuropsychiatric diseases [49]. Finally, PUFAs have anticancer activities during cancer development [50]. 

The PUFA/SFA and PUFA n-6/n-3 ratios, as well as AI, TI, and h/H index, are commonly used parameters to judge product nutritional value and the healthiness of intramuscular fat for human consumption. The following values of these parameters are recommended in human diets [15]: PUFA/SFA > 0.45, n-6/n-3 < 4, AI < 1, and TI < 0.5. With regard to the h/H index, nutritionally higher values are considered more beneficial for human health [15]. In our fish, the PUFA/SFA ratios were greater than 0.97, while the n-6/n-3 ratios were lower than 0.33. The AI and TI did not exceed 0.68 and 0.32, respectively. The h/H indices were >2 except for muksun, which also had less favorable nutritional indices in comparison to other fish species indicating a lower prohealth value of flesh obtained from *Coregonus muksun*. In general, all fish species analyzed can be recommended for human consumption. 

Most indigenous communities of the YNAO have elements of both a traditional and Western (or urban) lifestyle. Within our study area, the consumption of traditional fish has decreased by 37–50% in the past decade [2,51] due to a rapid westernization of lifestyles and a decline in fish stocks as a result of overexploitation of natural populations. In particular, this process is strongly expressed for muksun. Our data regarding the fatty acid composition of local salmonid fish may help in choosing the best alternative for *Coregonus muksun*. We found that the fatty acid profile of this species had the lowest dissimilarity with the least cisco, and the latter species, therefore, can be recommended as a replacement for muksun in the traditional diet.

## 5. Conclusions

The fatty acid profiles of four salmonid fish inhabiting water bodies of the Gydan Peninsula were assayed and compared with each other for the first time. Within the study site, the fatty acid signatures depended on fish species and the ontogenetic stages (in the case of Arctic cisco). Arctic charr demonstrated the highest concentrations of fatty acids, followed by adult Arctic cisco, muksun, least cisco, and juvenile Arctic cisco. There were significant spatial variations in the fatty acid composition among the Arctic, subarctic, and temperate populations of the species studied, most likely associated with different temperature regimes and feeding habits. All fish species have good nutrition quality in terms of high levels of essential fatty acids, optimal n-6/n-3 ratios, low atherogenic and thrombogenic indices, and relatively high h/H indices. Our data may have important implications for food policy and food security with regard to indigenous communities of northwestern Siberia.

## Figures and Tables

**Table 1:** The basic biological and sampling information on fish species collected for biochemical analyses from Russian Arctic water bodies.

Species Name	Common Name	Water Body	N	Month	L, cm	W, kg
*Coregonus autumnalis* (adult)	Arctic cisco	Gyda River	9	August	25–29	1.35–1.6
*Coregonus autumnalis* (juvenile)	Arctic cisco	Gyda River	9	August	11–12	0.22–0.24
*Coregonus sardinella*	Least cisco	Gyda River	9	August	12	0.14–0.15
*Coregonus muksun*	Muksun	Gyda River, Gyda Bay	9	September	48–51	1.45–1.5
*Salvelinus alpinus*	Arctic charr	Gyda River	9	September	39	1.35–1.41

Note. N—sample size, L—body length, W—body weight.

**Table 2:** Fatty acid composition in the flesh of four salmonid fish from the Gyda River and Gydan Bay.

Fatty Acid	Concentration, μg g^−1^					% of Total Fatty Acids				
	CAA	CAJ	CS	CM	SA	CAA	CAJ	CS	CM	SA
C6:0	0.3 ^a^	0.9 ^a^	5.5 ^b^	4.7 ^b^	6.5 ^c^	<0.01 ^a^	0.01 ^b^	0.05 ^c^	0.03 ^d^	0.02 ^e^
C8:0	1.4 ^a^	5.4 ^b^	5.6 ^b^	6.2 ^b^	5.9 ^b^	0.01 ^a^	0.07 ^b^	0.05 ^c^	0.04 ^c^	0.02 ^d^
C9:0	1.5 ^a^	3.6 ^a^	3.4 ^a^	3.9 ^a^^b^	6.8 ^b^	0.01 ^a^	0.05 ^b^	0.03 ^c^	0.03 ^c^	0.02 ^ac^
C10:0	2.9 ^a^	7.5 ^b^	7.4 ^b^	8.2 ^b^	11.5 ^c^	0.01 ^a^	0.10 ^b^	0.07 ^c^	0.06 ^c^	0.04 ^d^
C11:0	1.2 ^a^	1.2 ^a^	1.1 ^a^	1.0 ^a^	2.8 ^b^	0.01 ^a^	0.02 ^b^	0.01 ^a^	0.01 ^a^	0.01 ^a^
C12:0	75.2 ^a^	52.6 ^b^	63.4 ^c^	69.8 ^a^	71.1 ^a^	0.37 ^a^	0.70 ^b^	0.56 ^c^	0.51 ^c^	0.26 ^d^
C13:0	37.8 ^a^	54.3 ^b^	55.1 ^b^	68.3 ^c^	55.1 ^b^	0.19 ^a^	0.73 ^b^	0.49 ^ab^	0.49 ^ab^	0.20 ^a^
C14:0	1190 ^a^	206 ^b^	418 ^c^	758 ^d^	1050 ^e^	5.89 ^a^	2.76 ^b^	3.69 ^c^	5.48 ^a^	3.78 ^c^
C15:0	133 ^a^	61.3 ^b^	73.8 ^c^	108 ^a^	133 ^a^	0.66 ^a^	0.82 ^b^	0.65 ^a^	0.78 ^b^	0.48 ^c^
C16:0	3550 ^a^	1720 ^b^	2280 ^c^	2970 ^d^	5400 ^e^	17.6 ^a^	23.1 ^b^	20.2 ^ab^	21.5 ^b^	19.5 ^a^
C17:0	87.3 ^a^	37.7 ^b^	43.1 ^b^	89.7 ^a^	89.8 ^a^	0.43 ^ab^	0.50 ^a^	0.38 ^b^	0.65 ^c^	0.32 ^b^
C18:0	547 ^a^	391 ^b^	556 ^a^	698 ^c^	1590 ^d^	2.72 ^a^	5.23 ^bc^	4.91 ^b^	5.05 ^b^	5.74 ^c^
C20:0	34.0 ^a^	11.7 ^b^	11.7 ^b^	21.2 ^c^	42.0 ^d^	0.17 ^a^	0.16 ^a^	0.10 ^b^	0.15 ^a^	0.15 ^a^
C21:0	3.1 ^a^	4.9 ^a^	10.8 ^b^	1.9 ^c^	2.8 ^a^	0.02 ^a^	0.07 ^b^	0.10 ^c^	0.01 ^a^	0.01 ^a^
C22:0	12.9 ^a^	55.0 ^b^	30.3 ^c^	53.1 ^b^	47.6 ^b^	0.06 ^a^	0.73 ^b^	0.27 ^c^	0.38 ^d^	0.17 ^e^
C23:0	3.0 ^a^	2.1 ^a^	1.9 ^a^	3.1 ^a^	11.9 ^b^	0.01 ^a^	0.03 ^b^	0.02 ^a^	0.02 ^ab^	0.04 ^c^
C24:0	5.4 ^a^	5.3 ^a^	4.5 ^a^	8.8 ^b^	9.0 ^b^	0.03 ^a^	0.07 ^b^	0.04 ^a^	0.06 ^b^	0.03 ^a^
C14:1n5t	0.6	-	-	-	-	<0.01	-	-	-	-
C14:1n5c	26.8 ^a^	6.7 ^b^	9.3 ^c^	13.3 ^d^	31.8 ^e^	0.13 ^a^	0.09 ^b^	0.08 ^b^	0.10 ^b^	0.11 ^c^
C15:1	1.4 ^a^	0.5 ^b^	0.7 ^c^	0.5 ^b^	0.5 ^b^	0.01 ^a^	0.01 ^a^	0.01 ^a^	<0.01 ^b^	<0.01 ^b^
C16:1n7t	42.7 ^a^	0.8 ^b^	0.8 ^b^	0.8 ^b^	-	0.21 ^a^	0.01 ^b^	0.01 ^b^	0.01 ^b^	-
C16:1n7c	2670 ^a^	350 ^b^	942 ^c^	1370 ^d^	2810 ^a^	13.3 ^a^	4.69 ^b^	8.32 ^c^	9.94 ^d^	10.2 ^e^
C17:1	25.1 ^a^	1.0 ^b^	2.0 ^b^	2.1 ^b^	2.9 ^b^	0.12 ^a^	0.01 ^bc^	0.02 ^c^	0.02 ^c^	0.01 ^b^
C18:1n9t	14.8 ^a^	13.0 ^a^	12.4 ^a^	31.7 ^b^	34.6 ^b^	0.07 ^a^	0.17 ^b^	0.11 ^c^	0.23 ^d^	0.12 ^c^
C18:1n9c	3750 ^a^	850 ^b^	1390 ^c^	2460 ^d^	6340 ^e^	18.6 ^a^	11.4 ^b^	12.3 ^b^	17.8 ^a^	22.9 ^c^
C20:1	262 ^a^	48.9 ^b^	82.0 ^c^	157 ^d^	324 ^e^	1.30 ^a^	0.65 ^b^	0.72 ^b^	1.14 ^c^	1.17 ^c^
C22:1	21.9 ^a^	3.1 ^b^	7.0 ^b^	26.2 ^a^	44.8 ^c^	0.11 ^a^	0.04 ^b^	0.06 ^b^	0.19 ^c^	0.16 ^d^
C24:1	49.0 ^a^	10.0 ^b^	20.8 ^c^	80.4 ^d^	125.7 ^e^	0.24 ^a^	0.13 ^b^	0.18 ^c^	0.58 ^d^	0.45 ^e^
C18:2n6t	4.8 ^a^	1.0 ^b^	-	-	-	0.02 ^a^	0.01 ^b^	-	-	-
C18:2n6	697 ^a^	168 ^b^	255 ^c^	224 ^b^	757 ^d^	3.46 ^a^	2.24 ^b^	2.26 ^b^	1.62 ^c^	2.73 ^d^
C18:3n3	948 ^a^	179 ^b^	281 ^c^	170 ^b^	702 ^d^	4.71 ^a^	2.38 ^b^	2.48 ^b^	1.23 ^c^	2.53 ^b^
C18:3n6	8.5 ^a^	19.7 ^a^	108.8 ^b^	100.9 ^b^	15.9 ^a^	0.04 ^a^	0.26 ^b^	0.96 ^c^	0.73 ^d^	0.06 ^a^
C20:2n6	143 ^a^	38.0 ^b^	66.1 ^c^	39.4 ^b^	142 ^a^	0.71 ^a^	0.51 ^b^	0.58 ^c^	0.29 ^d^	0.51 ^b^
C20:3n6	36.6 ^a^	9.2 ^b^	10.1 ^b^	30.0 ^c^	71.9 ^d^	0.18 ^a^	0.12 ^b^	0.09 ^c^	0.22 ^d^	0.26 ^e^
C20:4n6	564 ^a^	260 ^b^	316 ^b^	371 ^bc^	669 ^d^	2.80 ^a^	3.46 ^b^	2.79 ^a^	2.69 ^a c^	2.41 ^c^
C22:2n6	10.9 ^a^	3.4 ^b^	1.5 ^c^	6.1 ^d^	35.1 ^e^	0.05 ^a^	0.04 ^a^	0.01 ^b^	0.04 ^a^	0.13 ^c^
C20:5n3	2110 ^a^	755 ^b^	1240 ^c^	1050 ^c^	1200 ^c^	10.5 ^a^	10.0 ^a^	10.9 ^a^	7.58 ^b^	4.34 ^c^
C22:6n3	2480 ^a^	1900 ^a^	2680 ^b^	1960 ^a^	4310 ^c^	12.3 ^a^	25.2 ^b^	23.6 ^b^	14.2 ^a^	15.6 ^a^
C20:3n3	95.1 ^a^	23.4 ^b^	35.2 ^c^	21.2 ^a^	155 ^d^	0.47 ^a^	0.31 ^b^	0.31 ^b^	0.15 ^c^	0.56 ^d^
C22:4n6	36.5 ^a^	9.1 ^b^	11.7 ^b^	54.2 ^c^	150 ^d^	0.18 ^a^	0.12 ^b^	0.10 ^c^	0.39 ^d^	0.54 ^e^
C22:3n3	2.1 ^a^	0.6 ^b^	-	-	-	0.01 ^a^	0.01 ^a^	-	-	-
C22:5n6	189 ^a^	101 ^b^	132 ^b^	146 ^c^	448 ^d^	0.94 ^a^	1.34 ^b^	1.17 ^ab^	1.06 ^a^	1.62 ^c^
C22:5n3	273 ^a^	129 ^b^	154 ^b^	635 ^c^	809 ^d^	1.36 ^a^	1.71 ^b^	1.36 ^a^	4.59 ^c^	2.92 ^d^
∑SFA	5680 ^a^	2620 ^b^	3580 ^c^	4880 ^d^	8530 ^e^	28.2 ^a^	35.1 ^b^	31.6 ^ab^	35.3 ^b^	30.8 ^ab^
∑MUFA	6870 ^a^	1280 ^b^	2470 ^c^	4150 ^d^	9720 ^e^	34.1 ^a^	17.2 ^b^	21.8 ^c^	30.0 ^d^	35.1 ^a^
∑PUFA	7600 ^a^	3600 ^b^	5290 ^c^	4800 ^c^	9470 ^d^	37.7 ^a^	47.7 ^b^	46.7 ^b^	34.7 ^a^	34.2 ^a^
Total	20,200 ^a^	7510 ^b^	11,300 ^c^	13,800 ^d^	27,800 ^e^	100	100	100	100	100
∑n-3	5910 ^a^	2990 ^b^	4380 ^c^	3830 ^b^	7180 ^d^	29.3 ^a^	39.6 ^b^	38.7 ^b^	27.7 ^a^	25.9 ^a^
∑n-6	1690 ^a^	609 ^b^	901 ^c^	972 ^c^	2290 ^d^	8.39 ^a^	8.11 ^a^	7.96 ^a^	7.03 ^b^	8.26 ^a^
n-6/n-3	0.29 ^a^	0.21 ^b^	0.21 ^b^	0.25 ^c^	0.32 ^a^	-	-	-	-	-
n-3/n-6	3.50 ^a^	4.87 ^b^	4.87 ^b^	3.94 ^c^	3.14 ^a^	-	-	-	-	-
AI	0.58 ^ab^	0.54 ^a^	0.52 ^a^	0.68 ^b^	0.50 ^a^	-	-	-	-	-
TI	0.24 ^ac^	0.24 ^ac^	0.22 ^a^	0.32 ^bc^	0.29 ^c^	-	-	-	-	-
h/H	2.30 ^a^	2.23 ^a^	2.38 ^a^	1.89 ^b^	2.33 ^a^	-	-	-	-	-
∑PUFA/∑SFA	1.34 ^ab^	1.38 ^ab^	1.48 ^b^	0.98 ^c^	1.11 ^a^	-	-	-	-	-

Note. SFA—saturated fatty acids, MUFA—monounsaturated fatty acids, PUFA—polyunsaturated fatty acids, AI—atherogenic index, TI—thrombogenic index, h/H—ratio between hypocholesterolemic and hypercholesterolemic fatty acids. Fish species: CAA—*Coregonus autumnalis* (adult specimens), CAJ—*Coregonus autumnalis* (juvenile specimens), SA—*Salvelinus alpinus*, CM—*Coregonus muksun*, CS—*Coregonus sardinella*. Different superscript letters denote significant statistical differences (one-way ANOVA with Tukey HSD post hoc, *p* < 0.05).

## Data Availability

The data are available on request from the corresponding author.

## Supplementary Materials

The following supporting information can be downloaded at: https://www.mdpi.com/article/10.3390/ani12131643/s1, Table S1. Analysis of similarity (ANOSIM): pair-wise comparisons of fatty acid content in fish species in the Gyda River, Russian Arctic. Table S2. SIMPER results of fatty acid profile data in fish species in the Gyda Peninsula, Russian Arctic. Pair-wise SIMPER tests between fish species. Fatty acids are ranked from the most to the least significant driver of the total dissimilarity; Contib% indicates contributions to the total dissimilarity. Figure S1. Spatial variations in fatty acid profiles (%) of salmonid fish from different habitats. (a)—Arctic cisco *Coregonus autumnalis* (adult), (b)—least cisco *Coregonus sardinella*, (c)—muksun *Coregonus muksun*, (d)—Arctic charr *Salvelinus alpinus*. Sources: Gyda River (Arctic, Siberia)—present study; Yenisei River (sub-Arctic, Siberia)—Gladyshev et al. (2017); Lake Baikal (temperate zone, Siberia)—Nikiforova et al. (2020); Lake Sobachye (sub-Arctic, Siberia), least cisco—Gladyshev et al. (2017), Arctic charr—Gladyshev et al. (2022); Lake Pyasino (Arctic, Siberia)—Sushchik et al. (2020); Lake Tokko, Lake Leprindo (temperate zone, Siberia), Lake Ladoga (subarctic zone, Karelia)—Gladyshev et al. (2022).
