# Supplementary material for: Fatty Acid Content of Four Salmonid Fish Consumed by Indigenous Peoples from the Yamal-Nenets Autonomous Okrug (Northwestern Siberia, Russia)
Source: Animals (Basel). 2022 Jun 27;12(13):1643. doi: 10.3390/ani12131643 (PMC9264761; doi:10.3390/ani12131643)
Supplement: Supplementary file 1 [file animals-12-01643-s001.zip › animals-1760769-supplementary.pdf]

Supplementary Material

Fatty acid content of four salmonid fish consumed by indigenous peoples from the Yamal-Nenets Autonomous Okrug (northwestern Siberia, Russia)

Alexander G. Dvoretsky [1], Fatima A. Bichkaeva [2], Olga S. Vlasova [2], Sergei V. Andronov [3] and Vladimir G. Dvoretsky [1,*]

[1] Murmansk Marine Biological Institute of the Russian Academy of Sciences (MMBI RAS), 183010 Murmansk, Russia; ag-dvoretsky@yandex.ru (A.G.D.)
[2] N. Laverov Federal Center for Integrated Arctic Research of the Ural Branch of the Russian Academy of Sciences (FECIAR UrB RAS), 163000 Arkhangelsk, Russia; fatima@fciarctic.ru (F.A.B.)., olgawlassova@mail.ru (O.S.V.)
[3] National Medical Research Center for Rehabilitation of Balneology, Moscow 121099, Russia; sergius198010@mail.ru (S.V.A.)
* Correspondence: v-dvoretsky@yandex.ru

Table S1. Analysis of similarity (ANOSIM): pair-wise comparisons of fatty acid content in fish species in the Gyda River, Russian Arctic.

Group	Group	R-Statistic	P-level
Coregonus autumnalis (adult)	*Coregonus autumnalis* (juvenile)	1	0.001
Coregonus autumnalis (adult)	*Salvelinus alpinus*	1	0.001
Coregonus autumnalis (adult)	*Coregonus muksun*	0.506	0.002
Coregonus autumnalis (adilt)	*Coregonus sardinella*	1	0.001
Coregonus autumnalis (juvenile)	*Salvelinus alpinus*	1	0.001
Coregonus autumnalis (juvenile)	*Coregonus muksun*	0.603	0.001
Coregonus autumnalis (juvenile)	*Coregonus sardinella*	1	0.001
Salvelinus alpinus	*Coregonus muksun*	0.868	0.001
Salvelinus alpinus	*Coregonus sardinella*	1	0.001
Coregonus muksun	*Coregonus sardinella*	0.471	0.001

Table S2. SIMPER results of fatty acid profile data in fish species in the Gyda Peninsula, Russian Arctic. Pair-wise SIMPER tests between fish species. Fatty acids are ranked from the most to the least significant driver of the total dissimilarity, Contib% indicates contributions to the total dissimilarity.

**CAA**	**CAJ**	**CAA**	**SA**	**CAJ**	**SA**	**CAA**	**CS**	**CAJ**	**CS**
Av. Diss = 26.65		Av. Diss = 12.69		Av. Diss = 32.17		Av. Diss = 14.40		Av. Diss = 14.94	
FA	Contrib%	FA	Contrib%	FA	Contrib%	FA	Contrib%	FA	Contrib%
C16:1C	13.12	C18:1n9	11.21	C18:1n9	15.05	C18:1n9	12.56	C16:1C	13.37
C18:1n9	12.79	C18:0	10.2	C16:1C	10.24	C16:1C	10.97	C18:1n9	10.75
C14:0	8	C22:6n3	9.69	C16:0	9.54	C14:0	7.33	C20:5n3	8.12
C20:5n3	7.39	C16:0	8.49	C22:6n3	6.64	C18:3n3	7.29	C14:0	7.36
C16:0	7.23	C22:5n3	7.34	C18:0	6	C16:0	6.32	C16:0	7.1
C18:3n3	6.93	C20:5n3	7.06	C14:0	5.37	C20:5n3	5.74	C22:6n3	6.38
C18:2n6	5.36	C22:5n6	4.56	C22:5n3	5.11	C18:2n6	5.44	C18:3n3	5.2
C20:1	3.66	C16:1t	4.05	C18:2n6	4.34	C18:3n6	3.83	C18:2n6	4.27
C20:4n6	3.06	C22:4n6	3.83	C18:3n3	3.91	C20:1	3.72	C18:3n6	4.07
C22:6n3	2.63	C18:3n3	2.72	C22:5n6	3.33	C20:4n6	3.18	C18:0	3.05
C20:2n6	2.31	C24:1	2.59	C20:1	3.29	C16:1t	2.88	C20:1	2.91
C16:1t	2.24	C17:1	2.07	C20:4n6	2.91	C22:5n3	2.18	C20:4n6	2.37
C22:5n3	2.09	C22:0	2.03	C22:4n6	2.76	C20:2n6	2.02	C20:2n6	2.26
C20:3n3	1.96	C20:3n3	1.65	C24:1	2.4	C20:3n3	2.01	C22:0	1.89
C17:1	1.61	C22:2n6	1.62	C20:3n3	2.28	C17:1	1.87	C22:5n3	1.62
C24:1	1.52	C20:3n6	1.49	C20:5n3	2.17	C15:0	1.62	C22:5n6	1.58
C22:0	1.51	C14:0	1.38	C20:2n6	1.71	C20:3n6	1.5	C24:1	1.55
C22:5n6	1.5	C18:2n6t	1.37	C20:3n6	1.62	C17:0	1.49	C20:3n3	1.55

**SA**	**CS**	**CAA**	**CM**	**CAJ**	**CM**	**SA**	**CM**	**CS**	**CM**
Av. Diss = 21.19		Av. Diss = 15.80		Av. Diss = 17.38		Av. Diss = 17.32		Av. Diss = 11.94	
FA	Contrib%	FA	Contrib%	FA	Contrib%	FA	Contrib%	FA	Contrib%
C18:1n9C	15.14	C18:3n3	10.82	C18:1n9c	13.76	C18:1n9c	14.68	C18:1n9c	10.46
C16:0	9.47	C16:1c	8.97	C16:1c	12.37	C22:6n3	10.48	C22:5n3	10.31
C16:1C	7.23	C20:5n3	8.32	C22:5n3	9.35	C16:0	9.29	C16:1c	7.04
C18:0	6.69	C18:1n9c	7.21	C14:0	8.87	C16:1C	7.8	C22:6n3	6.07
C22:5n3	6.19	C18:2n6c	6.99	C16:0	8.76	C18:3n3	6.58	C14:0	6.05
C22:6n3	5.91	C22:5n3	5.22	C18:0	4.48	C18:0	6.57	C18:3n3	5.89
C14:0	3.93	C18:3n6	4.31	C24:1	3.9	C18:2n6c	6.14	C16:0	5.41
C18:2n6C	3.77	C14:0	4.27	C18:3n6	3.77	C22:5n6	4.45	C20:5n3	4.42
C22:5n6	3.76	C22:6n3	3.49	C20:1	3.74	C20:3n3	3.84	C24:1	3.36
C18:3n3	3.46	C20:2n6	3.47	C20:5n3	3.34	C20:4n6	3.22	C20:1	3.03
C22:4n6	3.38	C16:1t	3.42	C22:4n6	2.93	C18:3n6	2.96	C22:4n6	2.93
C20:1	3.01	C16:0	3.18	C22:6n3	2.53	C20:2n6	2.75	C18:2n6c	2.86
C20:4n6	2.78	C20:3n3	3.14	C22:1	2.28	C20:1	2.67	C18:0	2.52
C24:1	2.5	C20:4n6	2.76	C17:0	2.24	C22:4n6	2.4	C20:2n6	2.33
C20:3n3	2.33	C20:1	2.24	C20:4n6	2.13	C14:0	2.35	C17:0	2.02
C18:3n6	2.14	C22:0	2.23	C15:0	1.73	C22:2n6	1.69	C22:0	1.93
C20:3n6	1.91	C17:1	2.17	C20:3n6	1.65	C22:5n3	1.59	C20:3n3	1.87
C22:2n6	1.73	C18:0	1.85	C22:5n6	1.4	C20:3n6	1.47	C20:4n6	1.84

Note: CAA – *Coregonus autumnalis* (adult specimens), CAJ – *Coregonus autumnalis* (juvenile specimens), SA – *Salvelinus alpinus*, CM – *Coregonus muksun*, CS – *Coregonus sardinella*

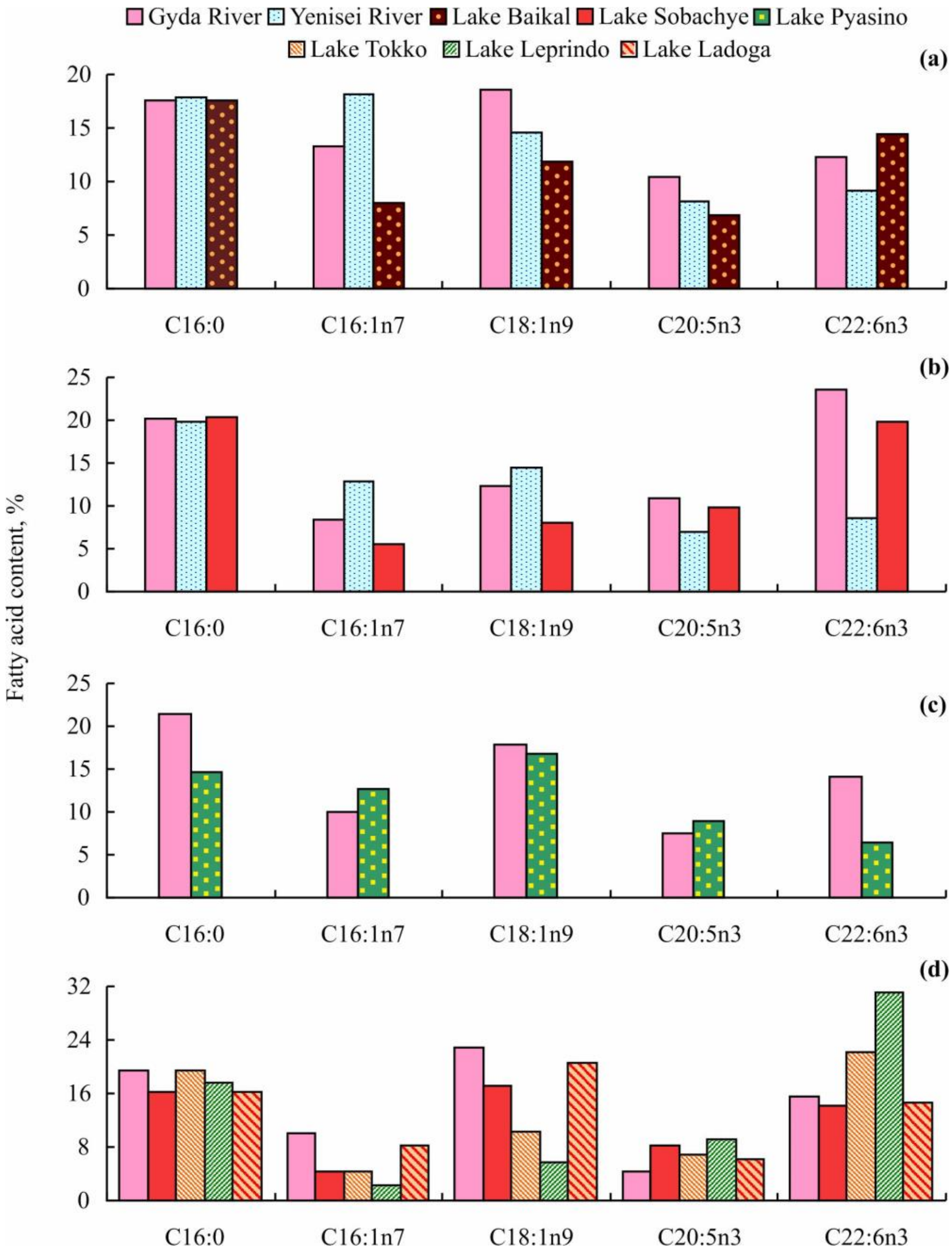

Figure S1. Spatial variations in fatty acid profiles (%) of salmonid fish from different habitats. **(a)** – Arctic cisco *Coregonus autumnalis* (adult), **(b)** – least cisco *Coregonus sardinella*, **(c)** – muksun *Coregonus muksun*, **(d)** – Arctic charr *Salvelinus alpinus*. Sources: Gyda River (Arctic, Siberia) – present study; Yenisei River (sub-Arctic, Siberia) – Gladyshev et al. (2017); Lake Baikal (temperate zone, Siberia) – Nikiforova et al. (2020); Lake Sobachye (sub-Arctic, Siberia), least cisco – Gladyshev et al. (2017), Arctic charr – Gladyshev et al. (2022); Lake Pyasino (Arctic, Siberia) – Sushchik et al. (2020); Lake Tokko, Lake Leprindo (temperate zone, Siberia), Lake Ladoga (subarctic zone, Karelia) – Gladyshev et al. (2022).

Gladyshev, M.I.; Sushchik, N.N.; Makhutova, O.N.; Glushchenko, L.A.; Rudchenko, A.E.; Makhrov, A.A.; Borovikova, E.A.; Dgebuadze, Y.Y. Fatty acid composition and contents of seven commercial fish species of genus *Coregonus* from Russian subarctic water bodies. *Lipids* **2017**, *52*, 1033–1044.

Gladyshev, M.I.; Makhrov, A.A.; Baydarov, I.V.; Safonova, S.S.; Golod, V.M.; Alekseyev, S.S.; Glushchenko, L.A.; Rudchenko, A.E.; Karpov, V.A.; Sushchik, N.N. Fatty acid composition and contents of fish of genus *Salvelinus* from natural ecosystems and aquaculture. *Biomolecules* **2022**, *12*, 144.

Nikiforova, A.; Zamaratskaia, G.; Pickova, J. Fatty acid composition of salted and fermented products from Baikal omul (*Coregonus autumnalis migratorius*) *J. Food. Sci. Technol.* **2020**, *57*, 595–605.

Sushchik, N.N.; Makhutova, O.N.; Rudchenko, A.E.; Glushchenko, L.A.; Shulepina, S.P.; Kolmakova, A.A.; Gladyshev, M.I. Comparison of fatty acid contents in major lipid classes of seven salmonid species from Siberian Arctic lakes. *Biomolecules* **2020**, *10*, 419.
